# A potential mechanism for compensation in the blue—yellow visual channel

**DOI:** 10.3389/fnhum.2013.00331

**Published:** 2013-07-03

**Authors:** Nicole T. Stringham, Dean Sabatinelli, James M. Stringham

**Affiliations:** ^1^Vision Sciences Laboratory, Department of Psychology, The University of GeorgiaAthens, GA, USA; ^2^Bioimaging Research Center, Department of Psychology, The University of GeorgiaAthens, GA, USA

**Keywords:** S-cone, koniocellular, visual cortex, color vision, psychophysics, electrophysiology, fMRI, temporal processing

## Abstract

Due to their unique contribution to human vision, the short (S)-wavelength sensitive cones, their anatomical projections and, more recently, the cortical representation of their function, have motivated intense scientific interest. The principal study of the visual channel associated with S-cone projections has been conducted using psychophysical, neurophysiological, and *ex vivo* anatomical techniques, whereas more recent research on the pathway has employed functional magnetic resonance imaging (fMRI). The purpose of this manuscript is to present a perspective regarding the means by which color signals within this visual channel are processed in the brain, namely how differences in short-wavelength light transmission caused by intraocular, pre-receptoral filtration are compensated for. Recent results from fMRI and psychophysical studies indicate the existence of a frequency-dependent signal amplification mechanism, whereby lower frequencies result in an amplification of S-cone signals. This finding could motivate a future research direction for determining the localization of blue—yellow color processing and neural compensation in the blue–yellow visual channel.

## Introduction

The validity and reliability of scientific results are strengthened greatly by a convergence of findings, derived using different methods. An excellent example of this convergence can be found in the study of human vision. Due largely to a desire to define the underlying mechanisms of perceptual experience, the early years of vision science were dominated by psychophysical inquiry. Physiological techniques for the study of response properties of retinal visual cells were developed and refined in the 1930s and 1940s (e.g., Hartline, 1940), enabling comparisons of psychophysics and cellular studies. The correspondence between these two methods was strong and demonstrable (e.g., Ratliff, 1965). As methodological sophistication progressed through the 1950s and 1960s, more specific information regarding the receptive field properties of mammalian retinal ganglion cells (Kuffler, 1953) and visual cortical neurons (Hubel and Wiesel, 1962, 1968) was generated. This information was found to relate in specific ways to perceptual experience. For example, the rudimentary basis for the detection of an edge could be explained as the result of cortical receptive field organization, where signals from center/surround retinal ganglion cells are compared; this ultimately leads to the enhancement of borders with contrast (i.e., edges).

More recently, non-invasive functional magnetic resonance imaging (fMRI) has allowed for reasonable localization of functional areas of cortex that correspond to psychophysical and physiological phenomena. For example, fMRI studies have begun to identify the cortical loci that support some of the processing involved in color and motion perception (Engel et al., 1997; Ahlfors et al., 1999; Wandell et al., 1999; Wade and Wandell, 2002; Liu and Wandell, 2005; Mullen et al., 2007, 2010; Wade et al., 2008; D'Souza et al., 2011); these results are largely consistent with the general findings from previous electrophysiological work (e.g., Zeki, 1980). With regard to specific aspects of color processing, the perceptual red/green (R/G) color system, served by the long (L)- and middle (M)-wavelength sensitive cones, has been well characterized by a number of fMRI studies (Engel et al., 1997; Wade and Wandell, 2002; Wade et al., 2008; Mullen et al., 2010). The characterization of specific functional loci that correspond to color processing for the blue/yellow (B/Y) color system [served by the short (S)-wavelength sensitive, and a combination of L/M cones], however, has proved somewhat challenging. Despite this, fMRI studies have revealed robust neural activation in V1 and extrastriate areas in response to putative S-cone-isolating stimuli (e.g., Engel et al., 1997; D'Souza et al., 2011). Nevertheless, the specific properties of this pathway and the neural loci to which it projects remain to be fully characterized. Indeed, in a recent review of human color processing, Shapley and Hawken (2011) highlight the lack of knowledge of the blue—yellow color processing channel as a pressing issue in color vision research by listing as one of the open questions of the last quarter century of research, “How are blue—yellow signals processed in the visual cortex?” Expanding on this, we address an additional question in our review, “What is the mechanism by which the B/Y color system compensates for short-wave light loss brought on by intraocular filters such as the crystalline lens and macular pigmentation (MP)?”

## Discrete visual channel for blue

For color vision to occur in humans, photons of light must enter the eye and be absorbed differentially by at least two of the three cone photoreceptor pigments. These pigments are embedded in photoreceptor outer segments and represent the first step in the conversion of light energy to a neural signal. The human retina has three types of broadly tuned, wavelength-/color-specific photoreceptors: long-wave (red), middle-wave (green), and short-wave (blue), or L-, M-, and S-cones, respectively. As the neural signal leaves the retina, signals from the three types of photoreceptors are combined into three independent cone-opponent channels, with two that correspond to color perception (R/G and B/Y; Hering, 1878/1964; Hurvich and Jameson, 1957). The R/G perceptual color system compares inputs from L- and M-cones, whereas the B/Y perceptual color system compares inputs from S- and a balanced combination of L- and M-cones (De Valois, 1965). These segregated cone-opponent signals continue to the lateral geniculate nucleus (LGN) of the thalamus (De Valois et al., 1966; Derrington et al., 1984) and primary visual cortex (V1), first synapsing in layer 4C-β (L- and M-cone-based parvocellular system; Hubel and Wiesel, 1972) and layers 2 and 3 of the cytochrome oxidase “blob” region of V1 (S-cone-based koniocellular system; Sincich and Horton, 2005), respectively. In addition to mediating R/G color perception, L- and M-cones also serve the parallel magnocellular visual stream, which mediates primarily perception of changes in brightness and motion (Sun et al., 2006).

The distinct R/G and B/Y color signal projections in visual cortex is one of many differences that distinguish the visual channel that processes blue from other visual channels. First, and perhaps most importantly, the evolutionary history of the B/Y channel suggests that it is phylogenetically ancient, predating the origin of mammals (Nathans et al., 1986; Jacobs, 1993). This lends support to the concept of the B/Y channel existing as a discrete visual system, largely independent of the L/M system. There are much data to support this conclusion. The anatomy of the B/Y channel is different from the L/M system in terms of morphology, spatial distribution, and projection layers in the retina and beyond. For example, S-cone photoreceptors and subsequent neural connections are under-represented by a factor of 10 relative to the L- and M-cone-based magno- and parvocellular visual streams: S-cones account for only roughly 8–10% of all cones in the retina (DeMonasterio et al., 1981; Ahnelt et al., 1987; Curcio et al., 1991), and are regularly, as opposed to randomly (in the case of L- and M-cones), distributed outside the foveola (DeMonasterio et al., 1981; Curcio et al., 1991). Furthermore, the synaptic connections that S-cones make in the inner layers of the retina are distinct—they appear to be served by a small-bistratified ganglion cell class (Dacey and Lee, 1994). Moreover, the signals that project from retina to LGN remain segregated and synapse in the intercalated regions between the magno- and parvo-cellular terminal layers, where they are referred to as koniocellular (Martin et al., 1997). As noted above, the geniculo-cortical projections of the koniocelluar system then synapse in a distinct region of the cytochrome oxidase “blobs” of the visual cortex, layers 2 and 3 (Hendry and Yoshioka, 1994), which appear to serve color perception (Livingstone and Hubel, 1984), and may contribute to form perception (Economides et al., 2011). Lastly, consistent with its projections to the blobs of V1, it appears that the B/Y system is distinct from the L/M system in terms of visual function, in that it serves primarily color perception and appears to contribute very little to brightness and motion perception (Sun et al., 2006; however, see Lee and Stromeyer, 1989; and Chatterjee and Callaway, 2002).

There is evidence that the visual system can independently adjust the signal balance within or between channels in order to render a stable color percept; in fact, individual arms of the opponent processes have been shown to be independent, in terms of contrast detection thresholds (Sankeralli and Mullen, 2001). In this way, adjustment of either the individual poles or of the balance of the B/Y channel could provide a means to compensate for short-wavelength light loss associated with the aging crystalline lens or MP Elsner et al., 1988; Werner et al., 2000; Stringham et al., 2006; Webster et al., 2010.

## The B/Y channel: isolation and compensation

As noted above, the S-cones feeding the B/Y system appear to serve color perception and do not appear to provide input to the luminance pathway. This idea, and the importance of accounting for MP, was elegantly demonstrated by Sun et al. (2006) in an electrophysiological study of the rhesus macaque retina. In order to isolate S-cones, Sun et al. (2006) accounted for the light filtering effects of MP and lens optical density on the relative spectral responsivities of the L-, M-, and S-cones by correcting for the lack of MP in the parafovea (the region of the retina tested); previous studies, e.g., Chatterjee and Callaway (2002), had used stimuli based on centrally derived spectral sensitivity functions that had not accounted for absorption (or lack thereof) of short-wavelength light by MP. Because all light that reaches the retina passes through the crystalline lens, an additional consideration in generating appropriate spectral conditions for S-cone isolating stimuli involves spectral correction for the filtering effect of the lens, which, due to denaturation of its structural proteins, yellows gradually with age. When the appropriate spectral corrections in the stimuli were made in order to facilitate S-cone isolation, Sun et al. (2006) found no functional S-cone contribution to the parasol ganglion cell types that form the magnocellular pathway. Previous studies (e.g., Lee and Stromeyer, 1989; Chatterjee and Callaway, 2002) had characterized an S-cone contribution, albeit minor, to the magnocellular pathway. In a second, control experiment, Sun et al. (2006) tested the same parafoveal retinal region, but used S-cone isolating stimuli based on cone fundamentals derived from foveal viewing (where MP is dense). As expected, these conditions gave rise to a false positive result of input to the magnocellular system. Based on these results, the S-cone input to the magnocellular pathway noted in previous studies would appear to be an artifact of the physical stimulus, and not a true neurophysiological phenomenon.

A color system such as that described above, with discrete channels that carry information about R/G and B/Y opponency, respectively, would be strongly dependent upon the within- and between-channel balance of visual information in order to ultimately render normal color vision. With regard to the B/Y channel, any intraocular factor that appreciably changes the amount of short-wavelength light reaching the already sparse S-cones would seemingly disrupt the relative balance within the B/Y color pathway. This would presumably lead to altered, or abnormal, color perception. Such factors include the two aforementioned pre-receptoral filters of short-wavelength light, the aging crystalline lens and MP. Inter-individual differences in short-wavelength filtration by the lens and MP can be quite large and can change dramatically over time. For instance, as we age, our intraocular lenses become yellowed, nearly doubling in short-wavelength light absorption from age 10 to 55 years (Pokorny et al., 1987). Those with yellowed lenses, however, do not report seeing the world with a “yellow tint.” In fact, these subjects exhibit normal color matches and report that the world looks exactly like it did when they were young (Elsner et al., 1988). Additionally, it has been shown that people with various levels of MP exhibit virtually flat S-cone sensitivity across the retina, despite MP's relatively high optical density in the fovea, and virtual absence in para- and perifoveal regions of the retina Werner et al., 2000; Stringham et al., 2006.

The yellow MP is composed of the carotenoids lutein and zeaxanthin and is obtained solely from dietary consumption of leafy-green vegetables (Humphries and Khacik, 2003). MP is found in rich concentration in the primate fovea (Snodderly et al., 1984a,b). By virtue of its yellow pigmentation and its location in the inner layers of the fovea, MP absorbs short-wavelength (blue) light before it reaches the foveal photoreceptors. Due to its dietary origin (and the variability in consumption of foods like leafy greens), levels of MP are extremely variable among subjects; some people (presumably those that consume greater quantities of the foods that contain L and Z) have enough MP to absorb nearly 90% of the blue light incident on the foveal region of the retina. By contrast, there are subjects with very little or no MP, which would result in transmission to the photoreceptors of nearly all the blue light incident on the fovea (Hammond and Caruso-Avery, 2000). One might expect, therefore, that the visual experience of someone with a high optical density of MP would differ greatly from someone with low MP optical density. As suggested above, and expanded upon below, this is apparently not the case.

Despite dramatic differences in MP optical density, subjects have been shown to maintain stable color appearance across the central retina. Hibino (1992), and subsequently Stringham and Hammond (2007), used a hue-cancelation technique wherein subjects indicated how much blue light (440 nm) was needed to mix with a yellow light (575 nm) in order to perceive white (neither blue nor yellow), as a function of retinal eccentricity. If there were no compensation for short-wavelength light loss via MP, the expected outcome of this procedure would be that more blue light would be needed to cancel the yellow in the center of the fovea (where MP is dense) vs. the parafovea. Results indicated, however, that B/Y color vision, despite major variations in the filtration of blue light via MP, is invariant across central retina. This was also true for a subject whose MP increased substantially, via supplementation with lutein, over six months (Stringham and Hammond, 2007). This subject's hue-cancelation values were assessed daily, and despite the changes in MP (and commensurate attenuation of short-wave light detected by the S-cones), the B/Y balance remained stable across the retina. Interestingly, the same result was not found for the R/G channel: in a fashion commensurate with their MP level, subjects required more “green” 501 nm energy (partially absorbed by MP) to cancel a “red” 600 nm light into yellow for central vs. parafoveal viewing. Furthermore, Werner et al. (2000) found that S-cone sensitivity was positively correlated with MP density and that, despite the presumed loss of S-cones with age (Haegerstrom-Portnoy, 1988), this effect was independent of age. The authors interpreted this as evidence of a visual gain mechanism that compensates for differential filtering of short-wave light by MP. Taken together, the results of studies presented above strongly suggest that an active, local compensatory mechanism is at work in the B/Y channel, and that the sensitivity of S-cone input appears to be adjusted to compensate for MP. This most certainly involves dynamic processes, perhaps throughout the visual system, that serve to regulate and balance inputs for the purpose of maintaining color uniformity. These dynamic processes begin at the level of the retina, where the visual system must first “normalize” its inputs, by maintaining some semblance of neural signal balance between the three cone types present in the normal human retina (e.g., von Kries adaptation; see West and Brill, 1982). It has been demonstrated (Neitz et al., 2002; Kulikowski et al., 2012), however, that von Kries adaptation is insufficient to explain color constancy and that cortical loci, such as area V4 (Zeki, 1980; Wild et al., 1985), inferior temporal cortex (Koida and Komatsu, 2007), parieto-temporal cortex (Rüttiger et al., 1999), and the fusiform color area and V1 (Barbur and Spang, 2008) are also involved in maintaining color constancy (see Foster, 2011, for a comprehensive review of color constancy). Indeed, Neitz et al. (2002) showed that the perception of unique yellow shifted significantly in subjects after long-term adaptation to either red- or green-tinted goggles. This effect points to a plastic, cortical mechanism at work to balance the L/M color system. Additionally, as noted above, when the S-cones are isolated via saturation of the L- and M-cones (e.g., Stringham et al., 2006), local retinal contrast cannot explain the dramatic increase in S-cone sensitivity required to offset absorption by MP in the central retina. Moreover, the hue-cancelation data described above from Hibino (1992) and Stringham and Hammond (2007) suggest a higher-order mechanism for compensation. Whereas color adaptation captures conceptually the idea of maintaining visual uniformity, it does not, with the exception of photopigment bleaching kinetics, directly implicate a specific neurophysiological mechanism. Similarly, the identification of a neural locus (e.g., area V4) involved in color processing informs potential compensatory mechanisms but does not, strictly speaking, identify one.

## Temporal processing: a potential mechanism for compensation

Based on the evidence discussed above, the primary function of the “on” response of S-cones in the B/Y channel is to signal the presence of short-wave light; this signal ultimately leads to the perception of “blue.” Given this, MP (which can absorb large fractions of short-wave light incident at or near the fovea) could severely disrupt the perception of blue for centrally viewed objects. This raises fundamentally important visual perception questions: (1) How does one perceive blue centrally, if much of the short-wavelength light is absorbed by MP and the crystalline lens before it reaches the sensory retina of the fovea? (2) Why does our overall color visual experience appear uniform across the visual field, despite large spatial changes in the amount, via MP, of short-wavelength light transmitted to the retina? The results presented above are suggestive of an active mechanism that “corrects” for perturbations in the spectral balance of visual inputs, perhaps calibrated to a standard light source, such as the sun. But where does the compensatory mechanism exist in the pathway, and how does it operate? Recent results from fMRI and psychophysical studies appear to provide valuable insight.

The results of several fMRI studies suggest that one critical way in which the B/Y channel is distinct from other visual streams is temporal processing. Mullen et al. (2008) measured hemodynamic activity in LGN and V1 during color perception tasks and found that LGN showed a robust response to R/G color contrast and a relatively weak response to B/Y contrast. At low presentation frequencies (2 Hz), however, the activation of the B/Y color pathway increased dramatically in V1 relative to LGN. The effect was found to be selective for temporal frequency—at 8 Hz, the B/Y signal change in V1 (compared to LGN) was much weaker. They attributed this to an amplification of S-cone signals between LGN and V1 when lower stimulus frequencies are used. Another fMRI study by Mullen et al. (2010) extended the findings of their previous work to demonstrate the same slow frequency–high activation relationship in more ventral areas in the visual stream, namely V4 and the ventral areas of V2 and V3. Again, this result suggests an amplification of blue channel pathway signal at some point between LGN and V1 (perhaps via geniculo-cortical feedback) or early in V1. Further support for the idea of S-cone signal amplification was provided by D'Souza and colleagues (2011). They report very compelling data, similar to Mullen et al.'s (2008, 2010), except that (perhaps due to higher stimulus contrasts used) the relationship between stimulus frequency and blood oxygen level dependent (BOLD) signal strength was stronger, and shown to be linear: in 2 Hz increments, from 12 Hz down to 2 Hz, lower-frequency modulation of the B/Y channel evoked a strongly significant, linear increase in V1 activity as well as ventral and temporal visual regions. Importantly, neither Mullen et al. (2008); Mullen et al. (2010) nor D'Souza et al. (2011) found this kind of relationship for the L/M cone-opponent system, which exhibited stable signal strength over the range of stimulus frequencies used. Taken together, the data of Mullen et al. (2008, 2010) and D'Souza et al. (2011) suggest an important role for stimulus frequency in determining S-cone signal strength in visual cortex. The reason for the differences between S-cone and L/M opponent temporal responses remains unclear. Perhaps more importantly, how do these differences manifest perceptually?

A recent psychophysical study of the temporal characteristics of the blue visual channel may complement the findings of Mullen et al. (2008, 2010) and D'Souza et al. (2011). Wood and Stringham (2012) tested S-cone critical flicker fusion thresholds (CFF) in subjects with a wide range of MP levels and found that, in a linear fashion, those with higher MP had significantly lower S-cone CFFs (indicative of slower temporal processing) for foveal viewing conditions. This was true despite controlling for the luminance loss brought on by MP. Additionally, the *reverse* was found for L/M-cone mediated CFFs: MP was significantly positively correlated to L/M-cone CFFs. Along with the fMRI data noted above, these psychophysical data suggest that compensation in the B/Y channel may be mediated via a signaling mechanism based on speed of visual processing, whereby slower frequencies lead to signal amplification. Mullen et al.'s (2008, 2010) and D'Souza et al.'s (2011) finding of a correlation between lower stimulus frequencies and greater amplification of signal in cortex for S-cone-isolating stimuli is consistent with this idea; the amplification of lower-frequency signals that occurs (presumably between LGN and V1 or early in V1) could represent the compensatory mechanism that yields uniform perception of blue across the visual field, despite the significant spatial differences in short-wavelength light transmission brought on by MP. To “close the loop” in the argument for frequency as a signal for compensation, a mechanistic link between the timing of stimulus frequency (used in fMRI studies), and the timing of the response of the system (CFF, used in psychophysics studies) must be established. A plausible link between the neural and perceptual phenomena could be temporal summation. Perhaps signals from S-cones are pooled temporally, which would increase signal strength at the expense of temporal resolution (and manifest as lower CFFs). This overall slowing of the S-cone signal may, however, not be enough to offset the dramatic loss of short-wave light in cases of high MP (where as much as 95% of available SW light at the fovea is absorbed before reaching the photoreceptors). This is where further signal amplification, perhaps between LGN and V1, may occur to compensate for short-wavelength light loss. In other words, the slower “front-end” system may serve as an indicator to visual processing further upstream to amplify the signal. The results of fMRI studies (Mullen et al., 2008, 2010; D'Souza et al., 2011) indicate that this may be the case in a linear fashion (similar to the MP–CFF relationship found in psychophysics).

## Conclusion

Psychophysical studies of S-cone sensitivity and perception of the color blue across the central retina indicate that, by virtue of a yet-to-be-discovered mechanism, the B/Y visual channel is adjusted to compensate for the absorption of short-wavelength light by MP and the crystalline lens. Recent psychophysical, physiological, and neuroimaging data, however, point to the possibility of stimulus frequency as a mechanism for B/Y channel gain control. To investigate this possibility, an experiment that assesses the level of signal amplification between LGN and V1, as a function of subjects' MP, could be conducted. Ideally, subjects with a wide range of MP (perhaps from 0 to 1.0 log unit of optical density) could be recruited. Additionally, within-subjects analysis could be included where data from central viewing (where MP is most dense) vs. peripheral viewing (where MP is optically negligible) are compared.

It has been demonstrated that, with careful control of stimulus conditions (i.e., accounting for MP), the B/Y channel can be isolated and studied independently of other visual channels. Importantly, via specific channel isolation, this type of work provides a means to vastly improve the sensitivity of recordings of the B/Y channel in cortex. This improvement provides a greater chance of determining, via non-invasive neuroimaging, how and where compensation for short-wavelength filters such as the crystalline lens and MP occur and, more generally, promises to improve our understanding of the processes by which color signals are processed in the human brain.

### Conflict of interest statement

The authors declare that the research was conducted in the absence of any commercial or financial relationships that could be construed as a potential conflict of interest.
